# Hereditary spherocytosis concomitant with JAK2V617F-positive primary myelofibrosis: a case report

**DOI:** 10.3389/fonc.2025.1665179

**Published:** 2025-10-17

**Authors:** Chi-E Qiu, Lei Lei, Guosong Jiang, Xiuqun Huang, Yadan Li

**Affiliations:** ^1^ Hematology Department, Northeast Yunnan Central Hospital, Zhaotong, Yunnan, China; ^2^ Department of Pulmonary and Critical Care Medicine, The First People’s Hospital of Zhaotong & The Zhaotong Affiliated Hospital of Kunming Medical University, Zhaotong, Yunnan, China; ^3^ Clinical Laboratory, The First People’s Hospital of Zhaotong & The Zhaotong Affiliated Hospital of Kunming Medical University, Zhaotong, Yunnan, China

**Keywords:** hereditary spherocytosis, primary myelofibrosis, JAK2V617F, comorbidity, myeloproliferative neoplasm

## Abstract

Hereditary spherocytosis (HS) is a genetic hemolytic disorder primarily characterized by hemolytic anemia, jaundice, splenomegaly, and frequent complications, including cholelithiasis, accompanied by the presence of spherocytes in the peripheral blood. This disorder predominantly follows an autosomal dominant inheritance pattern; however, certain cases exhibit an autosomal recessive mode of inheritance. HS is the most prevalent disorder associated with defects in the red blood cell membrane. Primary myelofibrosis (PMF), a chronic myeloproliferative neoplasm (MPN) characterized by splenomegaly resulting from extramedullary hematopoiesis, is associated with the *JAK2* V617F mutation. Currently, there are no documented instances of co-occurrence of HS and PMF in the literature. We report the case of a 37-year-old male who experienced recurrent abdominal distension and splenomegaly over the past decade, along with elevated platelet counts over the past nine years. The patient tested positive for the *JAK2V617F* mutation, and bone marrow smears revealed the presence of teardrop-shaped erythrocytes. Peripheral blood smears indicated the presence of approximately 20% of spherocytes. The morphology of the bone marrow biopsy specimen was consistent with an MPN, classified as MF-2 grade. The highly specific eosin-5’-maleimide binding assay demonstrated a reduced mean fluorescence intensity of 25.73%. The patient was managed with aspirin and ruxolitinib and continued to be monitored through follow-up evaluations.

## 1 Introduction

Hereditary spherocytosis (HS) is an inherited hemolytic disorder that can present at any age and is characterized by hemolytic anemia, jaundice, and splenomegaly, frequently complicated by gallstone formation, with the presence of spherocytes in peripheral blood smears. The incidence of HS is estimated to be approximately 1 in 2,000–3,000 individuals (1). Although specific incidence data for China are not available, HS is recognized as the most prevalent disorder of red blood cell membrane defects in the region (2). The clinical severity of HS varies, with the condition being predominantly inherited in an autosomal dominant manner, although certain cases demonstrate autosomal recessive inheritance (3). Diagnosis is primarily based on clinical presentation, family history, and laboratory evaluations, including peripheral blood spherocyte (PBS) count and the osmotic fragility test (OFT). Nevertheless, due to atypical clinical presentations in certain patients, the absence of family history, the lack of definitive diagnostic thresholds for PBS count, and suboptimal sensitivity and specificity of the OFT, there are significant rates of missed and misdiagnosed cases associated with HS (2).

Primary myelofibrosis (PMF) is a type of chronic myeloproliferative neoplasm (MPN) characterized by an insidious onset, often marked by significant splenomegaly, prominent hyperplasia of megakaryocytes and granulocytes in the bone marrow, reactive fibroconnective tissue deposition, and extramedullary hematopoiesis. Peripheral blood exhibits nucleated red blood cells and immature granulocytes, while bone marrow aspirate often yields a dry tap and displays hypoplasia. Its pathogenesis involves driver mutations in *JAK2*, *CALR*, and *MPL*, leading to the hyperactivation of the JAK-STAT signaling pathway, immune system dysregulation, and chronic inflammation. Immune system dysregulation is a major pathogenic mechanism in MF and serves as a key mediator of disease progression (4). The *JAK2* V617F mutation is one of the critical molecular markers for PMF diagnosis (5). Although not all patients with PMF carry this mutation, its high incidence makes it an important reference for diagnosis (6).

Splenomegaly is intricately linked to a range of hematologic disorders and frequently manifests as a clinical symptom of these conditions. As the largest lymphoid organ, the spleen is integral to blood filtration, the removal of senescent or abnormal blood cells, and the facilitation of immune responses (1). In the context of hematologic diseases, splenic enlargement can occur due to various pathological mechanisms. In cases of hemolytic anemia, increased destruction of red blood cells is the primary contributor to splenomegaly (7). This enhanced erythrocyte destruction, stemming from various etiologies, stimulates hematopoietic activity in the spleen, resulting in its enlargement (8). In myeloproliferative disorders, such as PMF, the normal hematopoietic function of the bone marrow is compromised, prompting the spleen to serve as a site for extramedullary hematopoiesis. This activation of extramedullary hematopoiesis leads to a significant increase in splenic size (9).

In this patient, the presence of splenomegaly was attributed to both HS and PMF. HS does not result in elevated white blood cell or platelet counts, while PMF seldom produces a substantial number of spherocytes. Given that a singular diagnosis could not account for the entirety of the patient’s clinical manifestations, additional diagnostic evaluation was performed using the highly specific eosin-5’-maleimide (EMA) binding assay. This assay confirmed the coexistence of HS and PMF.

## 2 Case description

**Figure 1:**
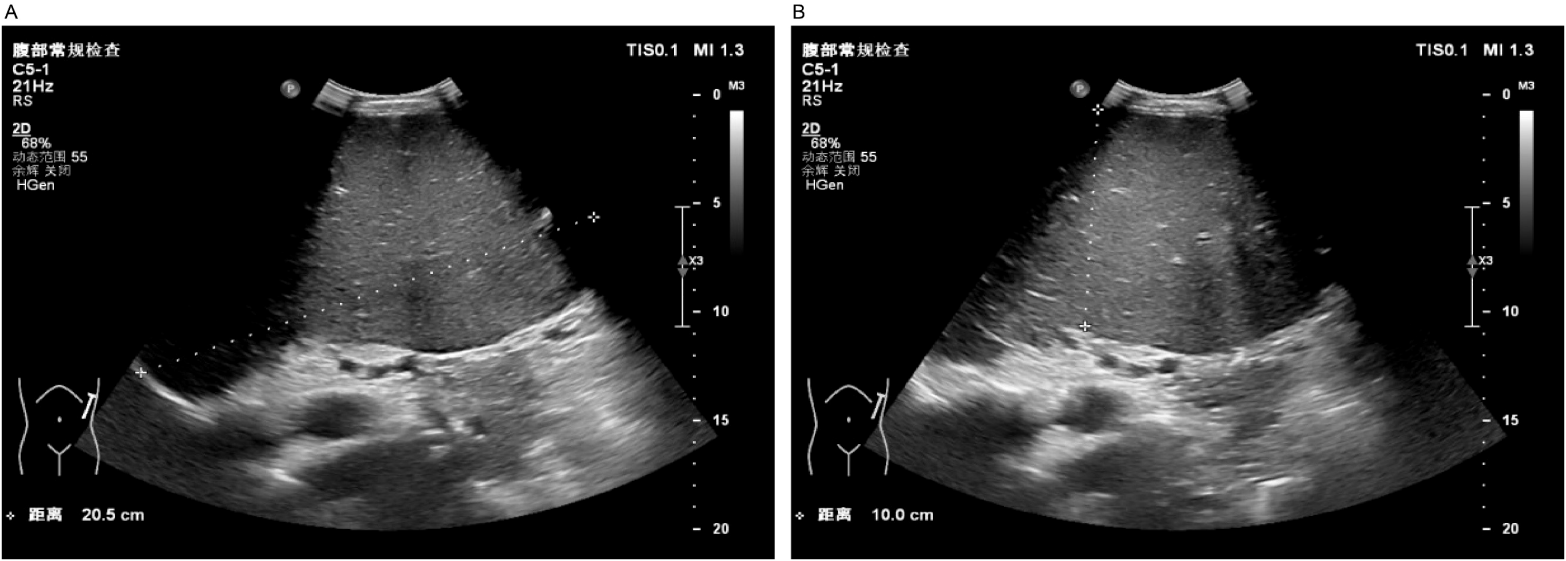
Abdominal ultrasound. Severe splenomegaly (approximately 10 cm in thickness, approximately 20 cm in the upper pole diameter, with the lower margin extending below the umbilical level and the medial border reaching the anterior midline). Panel **(A)** shows the craniocaudal diameter of the spleen, while Panel **(B)** illustrates the thickness of the spleen.

A 37-year-old male patient with a history of hypertension and no reported family history of anemia presented with a decade-long history of recurrent abdominal distension and splenomegaly, accompanied by elevated platelet counts persisting for nine years. Abdominal ultrasonography identified severe splenomegaly, characterized by a thickness of approximately 10 cm and a longitudinal diameter of approximately 20 cm, with the lower edge extending below the umbilical level and the medial border reaching the anterior midline. A patchy hypoechoic region was noted at the lower pole of the spleen, the etiology of which remains indeterminate but is most likely suggestive of an infarction (**Figure 1**).

**Figure 2:**
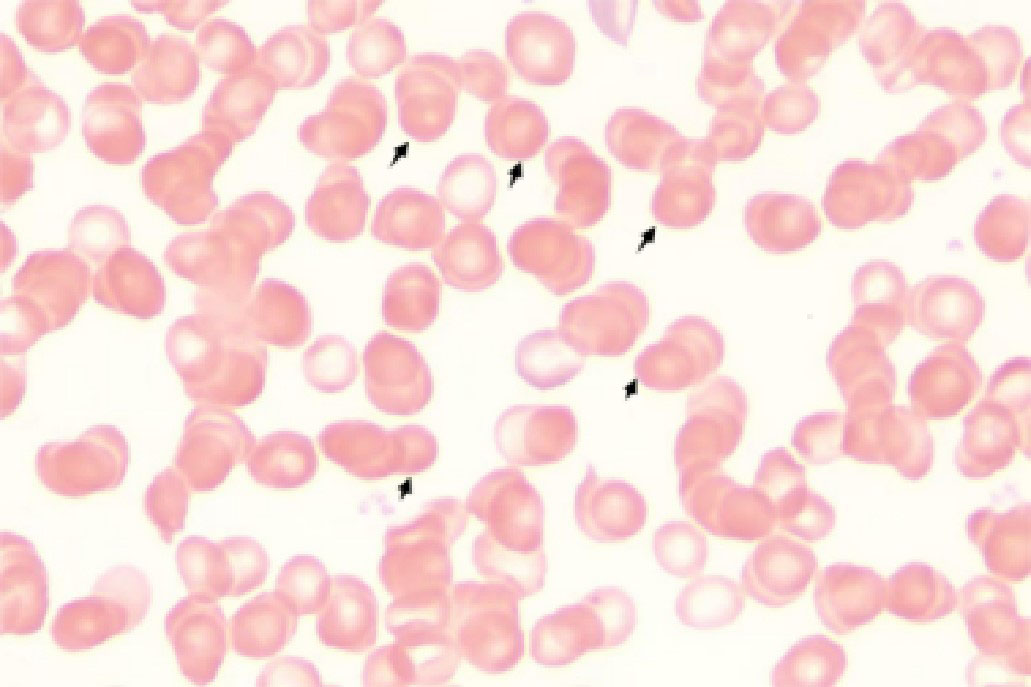
Peripheral blood smear. Approximately 20% spherocytes.

**Figure 3:**
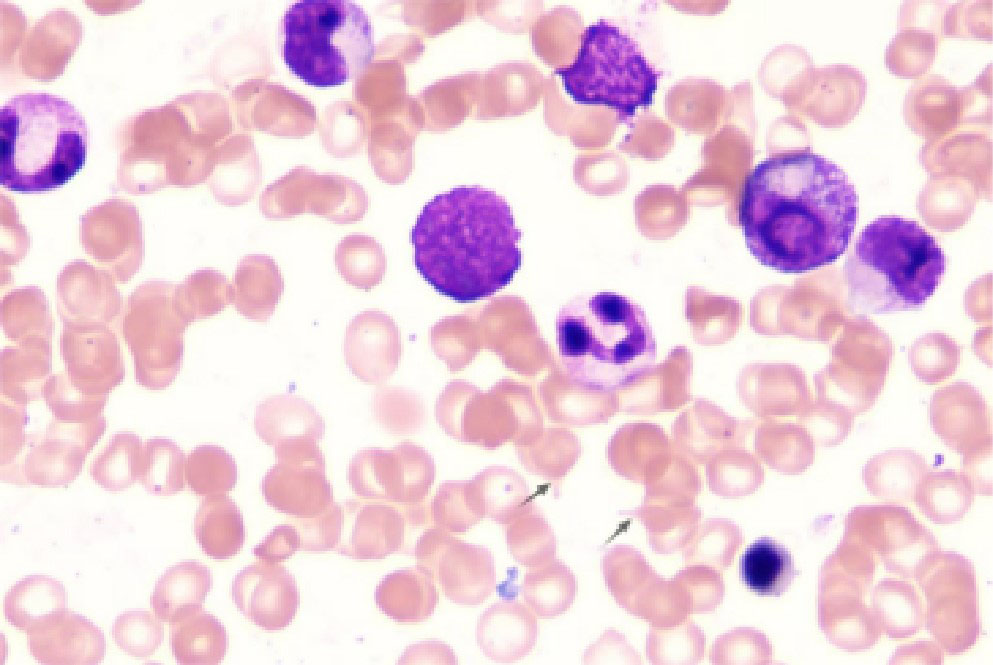
Bone marrow smear. Increased proliferation of granulocytic, erythroid, and megakaryocytic lineages in the bone marrow, with elevated platelets in both bone marrow and peripheral blood and the presence of teardrop-shaped red blood cells. Bone marrow biopsy morphology is consistent with myeloproliferative neoplasm (MF-2 grade).

**Figure 4:**
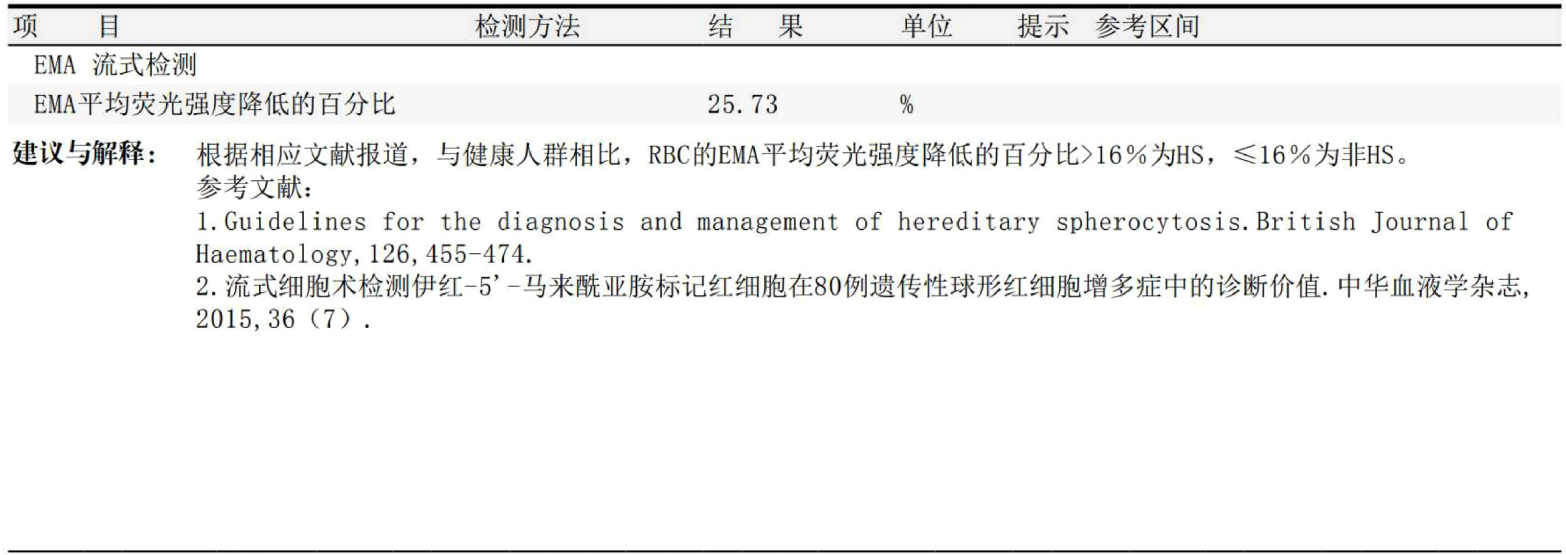
EMA binding test result. The excerpt from the laboratory report shows a mean fluorescence intensity reduction of 25.73%.

**Figure 5:**
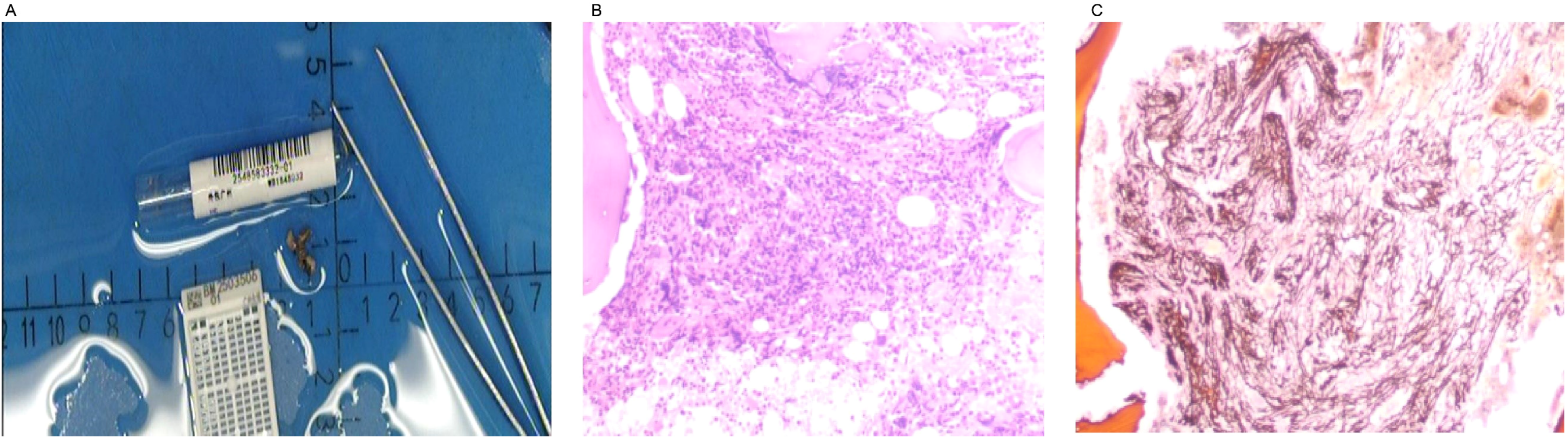
Bone marrow biopsy. The bone marrow biopsy morphology is consistent with myeloproliferative neoplasm (MF-2 grade). Panel **(A)** shows the bone marrow biopsy tissue specimen. Panel **(B)** displays the bone marrow biopsy tissue under the microscope. Panel **(C)** illustrates the reticulin fiber staining of the bone marrow biopsy tissue under the microscope.

Initial laboratory investigations revealed a platelet count of 772 × 10^9^/L, a white blood cell count of 15.40 × 10^9^/L, and a lactate dehydrogenase level of 315.00 U/L. The initial hemoglobin concentration was 101 g/L; however, during the clinical workup for his progressive symptoms, his hemoglobin level was documented to have declined to 74 g/L, indicating disease-related anemia. Peripheral blood smear analysis demonstrated hyperplasia across granulocytic, erythroid, and megakaryocytic lineages, with increased platelets in both the bone marrow and peripheral circulation, along with teardrop-shaped erythrocytes (**Figure 2**). Bone marrow smear analysis revealed the presence of approximately 20% spherocytes (**Figure 3**). The EMA mean fluorescence intensity exhibited a reduction of 25.73% (**Figure 4**). According to the pertinent literature, a reduction exceeding 16% compared to healthy individuals is indicative of HS, whereas a reduction of 16% or less is not (10). The osmotic fragility test yielded a negative result, as did the G-6-PD fluorescent spot test. The direct antiglobulin test results were also negative. Comprehensive screening for thalassemia gene mutations did not reveal any variants within the detectable range for both α- and β-thalassemia. Morphological analysis of the bone marrow biopsy was consistent with a myeloproliferative neoplasm of MF-2 grade (**Figure 5**). Allele-specific quantitative PCR identified the *JAK2* V617F mutation with an allele burden of 53.167%. Subsequent cytogenetic analysis of myeloproliferative disorders did not detect any additional genetic alterations, including mutations in *CALR*, *MPL*, *BCR-ABL1*, or other *JAK2* mutations, aside from *JAK2* V617F. Although myelofibrosis could account for the patient’s elevated white blood cell count, thrombocytosis, and splenomegaly, it did not explain the presence of 20% spherocytes in the peripheral blood. Therefore, a highly specific EMA binding test was conducted. The diagnoses were as follows: (1) HS; (2) PMF; (3) Hypertension. Regarding treatment and outcome, the patient was administered aspirin and ruxolitinib. However, due to a demanding work schedule, the patient did not consistently adhere to the prescribed medication regimen or attend regular follow-up appointments, leaving treatment efficacy under ongoing evaluation during subsequent follow-ups.

## 3 Discussion

This report describes the first documented case of HS with *JAK2* V617F-positive PMF. This case underscores the importance of peripheral blood smear examination in identifying the etiology of splenomegaly. Peripheral blood smears are instrumental in the diagnosis of hematologic disorders, as they provide detailed insights into cellular morphology (11). This case emphasizes the need for rigorous clinical reasoning when evaluating diseases with overlapping clinical manifestations to prevent potential diagnostic oversights.

This case illustrates the coexistence of HS PMF, characterized by marked splenomegaly, elevated leukocyte and platelet counts, mild anemia, and increased reticulocyte percentage. Peripheral blood analysis revealed numerous spherocytes that were not singularly attributed to either HS or PMF. A few patients with HS encounter diagnostic difficulties due to atypical clinical presentations, inconclusive laboratory results, or absence of familial history (3, 12). Both mean corpuscular volume (MCV) and mean corpuscular hemoglobin concentration (MCHC) were below the normal range in this case. Research indicates that only 8% of patients with HS exhibit subnormal MCV, while merely 32% have MCHC values above normal, suggesting that these parameters should not be used as definitive diagnostic criteria for HS (2). Blood smear analysis identified a significant presence of spherocytes and abnormal erythrocytes characterized by reduced size, spherical shape, and the absence of central pallor (13). These findings represent significant hematological abnormalities that may be indicative of multiple disorders. A thorough assessment of peripheral blood smears, along with pertinent laboratory tests, facilitates the precise diagnosis and implementation of suitable therapeutic interventions (14). HS continues to be one of the predominant causes of spherocyte formation.

In this case, the highly sensitive and specific EMA binding test (15) was used, significantly enhancing the reliability of the clinical diagnosis and confirming HS in the patient. This highlights the importance of blood smear examination in conjunction with bone marrow biopsy and genetic testing for the diagnosis of myeloproliferative disease to distinguish HS from PMF. Peripheral blood smears are crucial diagnostic tools for the assessment of myelofibrosis. Typically, peripheral blood smears in cases of myelofibrosis reveal characteristic abnormalities, including teardrop-shaped red blood cells, nucleated red blood cells, and immature granulocytes (16). In the current case, the peripheral blood smear demonstrated a marked increase in spherocytes and teardrop-shaped red blood cells, with an absence of immature granulocytes and nucleated red blood cells. The lack of immature granulocytes and nucleated red blood cells in myelofibrosis may be associated with the extent of fibrosis, disease progression, specific genetic mutations, and potential HS, warranting further investigation. Although peripheral blood smear examination is a vital tool for evaluating PMF, the absence of immature granulocytes and nucleated red blood cells should not be the sole criterion to exclude PMF (17). A definitive diagnosis of PMF necessitates comprehensive evaluation, including bone marrow biopsy, cytogenetic analysis, molecular biology testing, and clinical manifestations (18).

In this case, the patient had exhibited splenomegaly for more than a decade. Two prior bone marrow aspiration examinations were inconclusive in determining the etiology, as the patient reported a loss of medical records. A subsequent bone marrow examination conducted after a 9-year interval revealed the presence of teardrop-shaped red blood cells in the marrow, while a peripheral blood smear indicated approximately 20% spherocytes. Bone marrow biopsy demonstrated markedly active hyperplasia of nucleated cells, with an estimated hematopoietic capacity of 60%, accompanied by a diffuse and dense increase in reticulin fibers and extensive cross-linking, classified as MF-2 grade. Further genetic testing for MPN was performed, revealing a positive *JAK2* V617F mutation, and EMA flow cytometry indicated a 25.73% reduction in average fluorescence intensity. The concurrent diagnosis of overt PMF and HS in this patient was established through a comprehensive evaluation. The diagnosis of overt PMF was definitively confirmed as the patient’s findings fulfilled all three major and all four minor criteria of the 2022 World Health Organization (WHO) classification (19). Following this diagnosis, a formal risk stratification was conducted to guide therapeutic management, in line with the NCCN Clinical Practice Guidelines in Oncology for Myeloproliferative Neoplasms (Version 3.2022) (20). The patient’s clinical status met the criteria for two adverse factors within the Dynamic International Prognostic Scoring System (DIPSS): hemoglobin <100 g/L (2 points) and the presence of constitutional symptoms, manifested as severe abdominal distension (1 point). This yielded a total DIPSS score of 3, placing the patient in the Intermediate-2 (Int-2) risk category. For symptomatic patients with Int-2 risk PMF, the NCCN guidelines provide a Category 1 recommendation for JAK1/2 inhibitor therapy. Therefore, the initiation of ruxolitinib was a guideline-directed decision to address his significant disease burden, including massive splenomegaly and progressive anemia. A further diagnostic consideration was the confirmation of HS. We acknowledge that targeted gene sequencing of red cell membrane protein genes (*ANK1, SPTB, SLC4A1, SPTA1, EPB42*) is the gold standard for a definitive molecular diagnosis. This testing was recommended to the patient to confirm the diagnosis and clarify the inheritance pattern. However, the patient declined to undergo this investigation due to prohibitive out-of-pocket costs, a decision that was formally documented. The clinical diagnosis of HS remains robustly supported by the presence of 20% spherocytes on the peripheral blood smear and a definitively positive eosin-5’-maleimide (EMA) binding test. While the absence of genetic data is a limitation of this report, it did not alter the immediate therapeutic management, which was driven by his symptomatic, Intermediate-2 risk PMF. We plan to seek opportunities for this genetic analysis for the patient during future follow-up. Case reports have suggested that patients with HS may develop secondary myelofibrosis and acute monocytic leukemia (M5) following splenectomy (21). PMF is a hematopoietic clonal disorder of unknown etiology, and the relationship between PMF and HS remains to be elucidated. This hypothesis posits that defects in red blood cell membranes of HS result in elevated ATP consumption. This raises the question of whether prolonged ATP deficiency in the bone marrow predisposes individuals to driver mutations in *JAK2*, *CALR*, and MPL, thereby contributing to the development of myelofibrosis. The potential correlation between HS and PMF necessitates further investigation to determine whether this association is coincidental or indicative of an underlying relationship. When a singular theoretical framework fails to account for all observed phenomena, a comprehensive approach should be adopted to enhance diagnostic precision and prevent misdiagnosis or oversight. By emphasizing the morphology of peripheral blood smears, diagnostic and differential capabilities can be maximized, thereby significantly advancing hematological morphological diagnostics.

## Data Availability

The raw data supporting the conclusions of this article will be made available by the authors, without undue reservation.
